# Arterial involvement in Erdheim–Chester disease: A retrospective cohort study

**DOI:** 10.1097/MD.0000000000013452

**Published:** 2018-12-10

**Authors:** Michel Villatoro-Villar, Michael S. Bold, Kenneth J. Warrington, Cynthia S. Crowson, Gaurav Goyal, Mithun Shah, Ronald S. Go, Matthew J. Koster

**Affiliations:** a Division of Vascular Radiology; b Department of Diagnostic Radiology; c Division of Rheumatology; d Department of Health Sciences Research; e Division of Hematology, Mayo Clinic, Rochester, MN.

**Keywords:** Erdheim–Chester disease, non-Langerhans cell histiocytosis, vascular disease

## Abstract

Erdheim-Chester disease (ECD) is a rare histiocytosis of the “L” (Langerhans) group with multisystem involvement that can affect the large and medium-sized arteries mimicking vasculitis. Aortic involvement is common but the frequency and outcome of aortic branch vessel abnormalities are less well described.

Patients with ECD were retrospectively identified. Images containing information of arterial involvement within 6 months of diagnosis were considered baseline and compared to last follow-up studies. Two physicians independently reviewed the studies to evaluate for presence of abnormalities attributable to ECD. Age and sex-adjusted logistic regression models were used to examine associations between patient characteristics and vessel involvement at baseline.

Among a cohort of 64 patients with ECD, 63 had baseline imaging of vascular structures. ECD involvement of at least 1 segment of the aorta was observed in 56%. Abnormalities were also observed in aortic arch branches (26%), visceral branch arteries (40%), iliofemoral arteries (31%), coronary (5%), and pulmonary (3%) arteries. Perinephric fibrosis was strongly associated with the identification of abnormalities in the thoracic aorta (OR 4.92 [1.54, 15.75]; *P* *=* .007), abdominal aorta (OR 7.57 [2.28, 25.07]; *P* = .001) and visceral branch arteries (OR 6.05 [1.52, 24.03]; *P* = .01) but not pelvic/lower extremity arteries. Complete normalization of arterial abnormalities at follow-up was only observed in 9% or less of arterial segments involved at baseline.

Aortic and aortic branch vessel abnormalities are frequently observed in patients with ECD and are often asymptomatic. Partial and/or complete resolution of arterial findings is uncommon.

## Abstract 2

Supplemental Digital Content is available in the text

## 1 Introduction

Erdheim–Chester disease (ECD) is a rare histiocytosis of the “L” (Langerhans) group^[1]^, initially described in 1930.^[2]^ It is characterized by a multi-system xanthogranulomatous infiltration of CD68 (+)/CD1a (−) lipid-laden macrophages with a surrounding fibro-inflammatory stroma.^[3]^ Although bone and perinephric infiltration are the most common locations of lesioned tissue,^[4,5]^ periaortic infiltration is frequently seen and has been reported in 55% to 82% of cases.^[5–8]^

Despite its frequency, vascular involvement is rarely the predominant feature at presentation^[9]^ and can occur in the absence of respiratory and cardiac symptoms.^[8]^ As such, data regarding vascular involvement in case reports are often incomplete, overlooked, and underestimated.^[6]^ While periaortic involvement has been described in single-center cohorts^[5–8,10,11]^ limited information is present regarding the frequency and severity of the aortic branch vessels. Furthermore, few studies have described the evolution of aortic or branch vessel abnormalities over the course of disease.

The purpose of this study was to describe the frequency and distribution of aortic and branch vessel involvement at baseline and evaluate arterial outcomes at last follow-up in a large, single-institution cohort of patients with ECD.

## 2 Methods

### 2.1 Study design

This was a retrospective study of patients diagnosed with ECD at Mayo Clinic, Rochester, MN, USA between January 1, 1998 and July 31, 2016. The identification of patients with ECD based on clinical and histopathologic analysis has been previously reported.^[12]^ The study was approved by the Institutional Review Board at the Mayo Clinic, Rochester, Minnesota.

### 2.2 Imaging

Imaging studies including echocardiography, computed tomography (CT), computed tomography angiography (CTA), magnetic resonance imaging (MRI), magnetic resonance angiography (MRA), positron emission tomography (PET), vascular ultrasonography, and digital subtraction angiography were independently reviewed by a physician (MVV) and a radiologist (MSB). If disagreement in interpretation was present, consensus was reached by third physician review (MJK). Studies performed within 6 months of ECD diagnosis were considered baseline. Only patients with baseline and follow-up imaging were used for comparison of vascular outcomes.

### 2.3 Definitions

Arterial abnormalities of interest included ectasia, aneurysm, stenosis, occlusion, periarterial thickening and 2-deoxy-2-[fluorine-18]-fluoro-D-glucose (FDG) hypermetabolism. Ectasia was defined as a segment of abnormal dilation or distention ≤50% of normal arterial diameter for the territory evaluated, whereas designation of aneurysm was used if the dilation or distention exceeded 50%. Stenosis was defined as an abnormal narrowing (approximated as ≥25% luminal reduction) of an arterial segment due to non-atherosclerotic cause and occlusion defined as radiographic loss of patency. Periarterial thickening was defined as adventitial or periadventitial soft tissue attenuation greater than 2 mm in radial dimension. Arterial FDG uptake on PET or PET-CT was evaluated based on a 4 point visual scale with vascular uptake scores ≥2 (i.e., equal to or higher than liver uptake) considered as increased and scores of 0 and 1 (i.e., less than liver uptake) considered as negative.^[13]^

Cardiac involvement was assessed through presence of objective evidence of 1 or more of the following: pericardial effusion, pericardial thickening, myocardial thickening, right atrial pseudotumor, and non-atherosclerotic coronary artery thickening. Evaluation of retroperitoneal involvement included the presence of 1 or more of the following: periaortic (abdominal) thickening, perinephric and/or periureteral fibrosis and hydronephrosis.

### 2.4 Assessment of response

Findings were compared at baseline and last follow-up visit. Arterial abnormalities were evaluated radiographically to determine if the baseline findings progressed, resolved or partially resolved. Progression was defined as increase in the areas of arterial involvement, resolution (complete response) was defined as no evidence of arterial involvement in any of the follow-up imaging studies; partially resolved (partial response) was defined as improvement in the previously observed arterial abnormalities as noted by a ≥25% reduction of periarterial thickening or FDG uptake. Areas, where initial findings were normal at baseline but present at follow-up, were considered as newly involved.

### 2.5 Data collection

In addition to imaging characteristics, baseline data including demographics, initial presentation symptoms, co-morbidities, laboratory parameters, treatment and BRAF-V600E status were abstracted from direct medical chart review.

### 2.6 Statistical analysis

Descriptive statistics (means, percentages, etc.) were used to summarize the data. Comparisons between patients with negative and positive BRAF-V600E were performed using chi-square and rank sum tests. Age and sex-adjusted logistic regression models were used to examine associations between patient characteristics and vessel involvement at baseline. Analyses were performed using SAS version 9.4 (SAS Institute, Cary, NC).

## 3 Results

### 3.1 Baseline characteristics

A total of 64 patients were included in this study with baseline characteristics outlined in Table 1. Forty-four (69%) were male and 54 (84%) Caucasian. Median length of follow-up was 2.5 years, with the longest follow-up length covering 16.8 years.

**Table 1:**
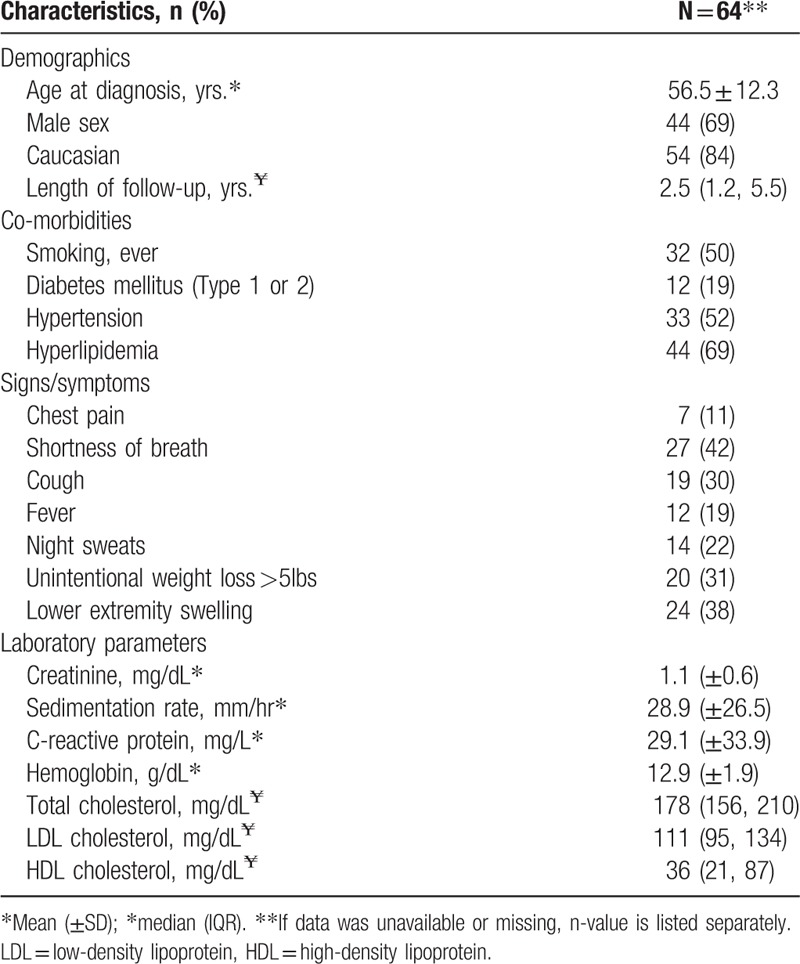
Baseline characteristics and presentation features of patients with Erdheim–Chester disease.

Cardiovascular risk factors were present in the majority of patients with 50% (n = 32) being current or former smokers, 52% (n = 33) hypertensive, and 69% (n = 44) with evidence of hyperlipidemia at baseline.

Symptomatic shortness of breath was present in 42% of patients; however, chest pain was infrequent (11%). Constitutional symptoms of fever, night sweats, and unintentional weight loss were relatively common and observed in 19%, 22%, and 31% of patients, respectively. Lower extremity swelling was present in 38% of cases.

Mean creatinine at diagnosis was 1.1 ± 0.6 but ranged between 0.4 to 4.6 mg/dL. The erythrocyte sedimentation rate was elevated (≥22 mm/hr males, ≥29 mm/hr females) in 47% of patients, but the overall mean for the cohort was modest at 28.9 ± 26.5. C-reactive protein was increased (≥8.0 mg/L) in the majority (81%) of patients with a mean of 29.1 ± 33.9 at baseline.

Imaging present at or within 6 months of diagnosis included 33 echocardiograms, 103 CTs (51 chest/thorax, 58 abdomen/pelvis and 4 lower extremity with runoff), 20 MRIs (11 chest/thorax and 9 abdomen/pelvis), 21 abdominal ultrasounds, 7 catheter-based coronary angiograms, and 25 PET/PET-CTs.

BRAF-V600E was evaluated in 22 of 64 patients and was positive by either immunohistochemistry or polymerase chain reaction (PCR) analysis in 12 patients, and negative in 10 patients. BRAF mutation was not significantly associated with cardiac, retroperitoneal or vascular involvement (*P* = .26, *P* = .93, and *P* = .84 respectively). No other baseline characteristics were significantly associated with BRAF positivity.

Treatment at baseline was not standardized. Among the 64 patients in the cohort, 8 did not receive any treatment during follow-up. For those receiving treatment, corticosteroids were the most frequently utilized first-line agent (n = 22) followed by cladribine (n = 10), methotrexate (n = 7), interferon (n = 5), radiation (n = 5), tamoxifen (n = 3), cyclophosphamide (n = 2) anakinra (n = 2), vinblastine (n = 2) and imatinib (n = 1). Two patients, both with BRAF mutations, received vemurafenib at baseline. Throughout follow-up, treatment was highly variable with 34 (53%) of patients requiring a second line, 22 (34%) a third line, 12 (19%) a fourth line, and 5 (8%) a fifth line of treatment. Overall, BRAF-inhibitors were utilized during the course of disease in 12 (19%) patients (10 BRAF-positive, 1 BRAF-negative, 1 non-tested).

## 4 Cardiac involvement

**Figure 1:**
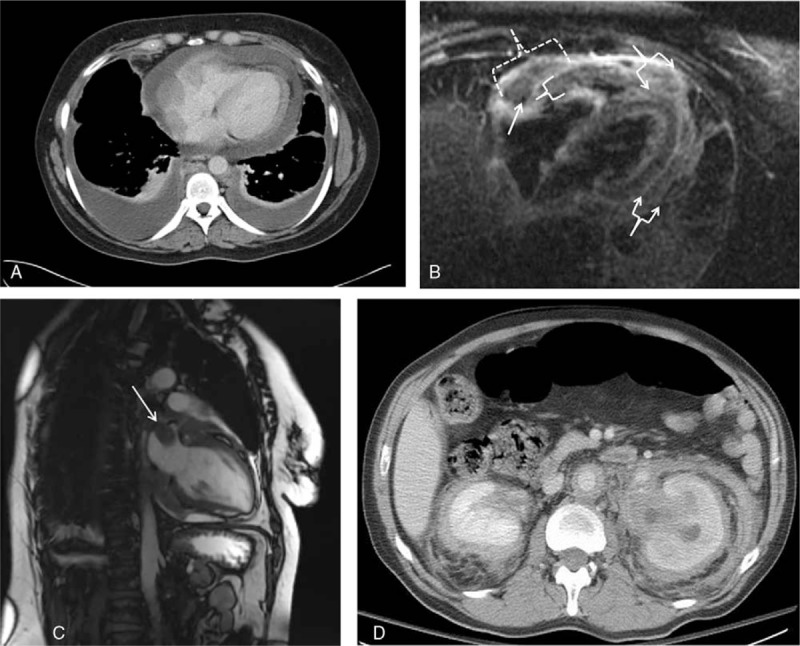
Cardiac and retroperitoneal involvement in Erdheim-Chester disease. Figure 1A: Circumferential pericardial thickening and effusion (contrast enhanced computed tomography, axial view). Figure 1B: Cardiac magnetic resonance imaging, 3 chamber triple inversion view, demonstrates pericardial thickening (arrowed brackets) and infiltration into the atrioventricular groove (dotted bracket) with circumferential encasement of the right coronary artery (arrow). Additionally, there is moderate myocardial thickening of the right ventricular free wall (bracket). Figure 1C: Right atrial pseudotumor [arrow] (cardiac magnetic resonance imaging, T1-weighted, sagittal view). Figure 1D: Bilateral perinephric fibrosis (“hairy kidney”) and circumferential abdominal periaortic thickening (contrast enhanced computed tomography, axial view).

Fifty-seven (89%) patients had 1 or more imaging evaluations of cardiac structures within the first 6 months of diagnosis. Cardiac involvement was observed in 35 (61%). Pericardial effusion was observed in 22/49 (45%), pericardial thickening in 14/45 (31%) and myocardial thickening in 17/45 (38%) patients [Fig. 1A and 1B]. Right atrial pseudotumor was detected in 6/45 (13%) [Fig. 1C] and non-atherosclerotic coronary artery thickening identified in 9/38 (24%) patients.

## 5 Retroperitoneal involvement

Baseline imaging of the abdomen (ultrasound, CT, MRI, and PET/CT) was available in 58 patients. Perinephric fibrosis was observed in 34 (59%) [Fig. 1D] and isolated periureteral fibrosis in only 7 (12%) patients. Hydronephrosis was seen in 17 patients, 10 of which had bilateral involvement; 11 patients were treated medically, unilateral ureteral stenting was performed in 2 patients and bilateral ureteral stenting in 4 patients because of severe obstructive uropathy present at diagnosis.

## 6 Arterial involvement

**Figure 2:**
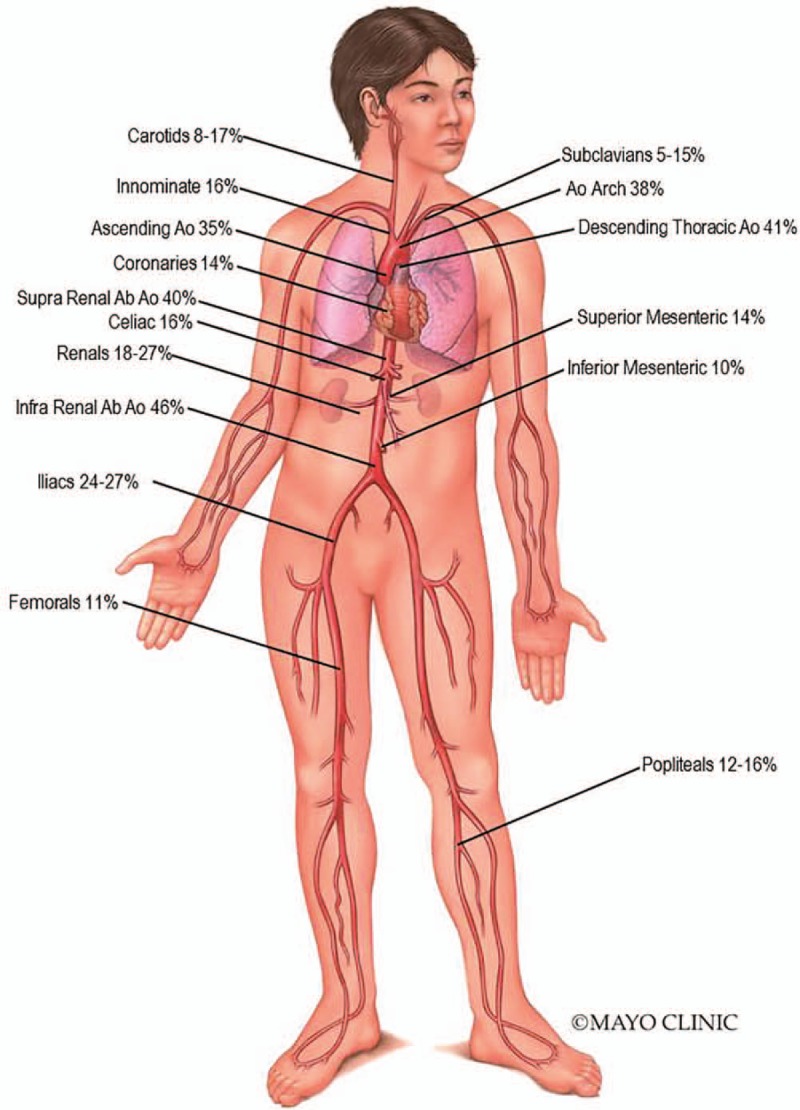
Arterial Map in Erdheim-Chester disease. Percentages show overall distribution of arterial involvement. Ao = Aorta; Ab = Abdominal.

Sixty-three patients (98%) had imaging of vascular structures performed within 6 months of baseline diagnosis. Characteristics of arterial abnormalities by regional vascular group are presented in Table 2 and summarily depicted in Figure 2.

**Table 2:**
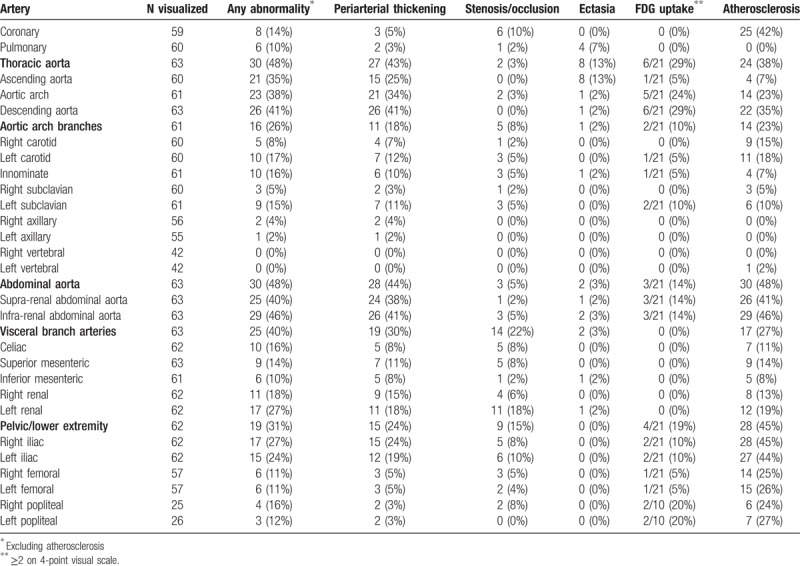
Arterial abnormalities present at baseline imaging in patients with Erdheim–Chester disease.

**Figure 3:**
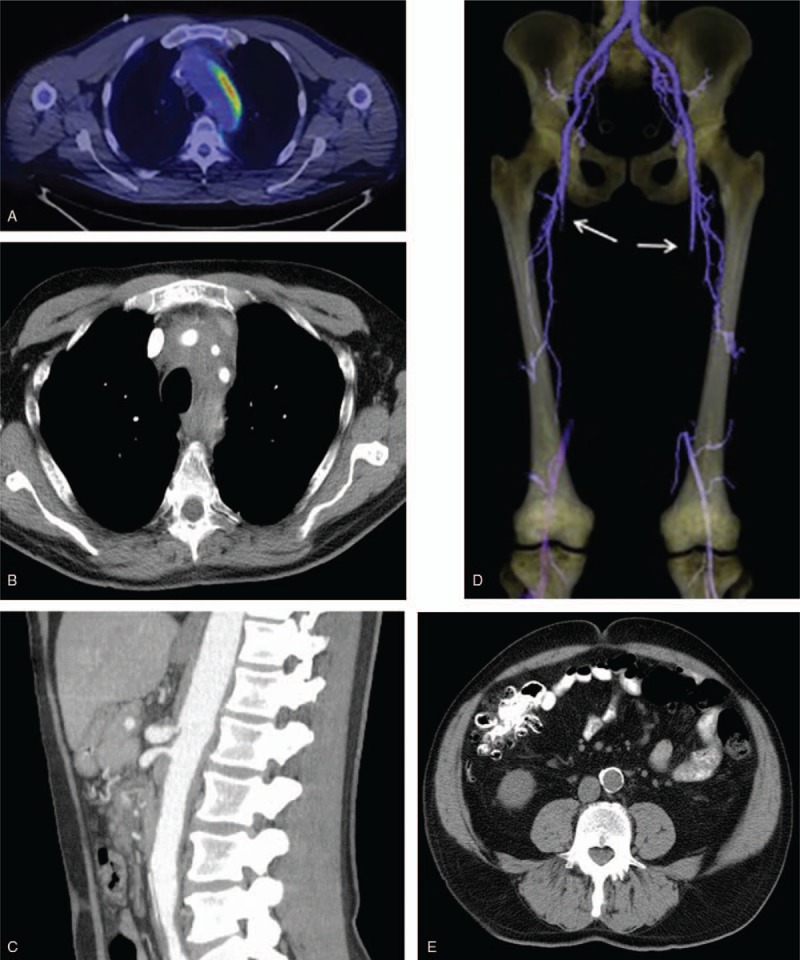
Arterial involvement in Erdheim-Chester Disease. Figure 3A: Asymmetric increased vascular hypermetabolism of the thoracic aorta (FDG PET/CT, axial fusion image). Figure 3B: Periadvential thickening of the left subclavian, left carotid and innominate arteries (contrast enhanced computed tomography angiography, axial view). Figure 3C: High grade stenosis with post-stenotic dilatation of the celiac artery and moderate stenosis of superior mesenteric artery (contrast enhanced computed tomography angiography, sagittal view). Figure 3D: Bilateral superficial femoral artery occlusion (arrows) with reconstitution at the level of the popliteal artery (3-dimensional maximum intensity projection, computed tomography angiography). Figure 3E: Dense atherosclerosis of the abdominal aorta (computed tomography angiography, sagittal view).

Aortic involvement with ECD was observed in at least 1 segment of the aorta in 56% of patients at baseline. The thoracic and abdominal aortic segments were the most commonly affected with 48% of patients, demonstrating at least 1 abnormality in each location. Among the aortic segments affected, the frequency of abnormalities seen was highest in the infra-renal abdominal aorta (46%), followed by descending thoracic aorta (41%), supra-renal abdominal aorta (40%), aortic arch (38%), and ascending aorta (35%). Periarterial thickening was the most frequently observed abnormality. At baseline, arterial aneurysm was rarely observed. In fact, no aortic aneurysms were seen and only one patient had evidence of post-stenotic aneurysm in the celiac artery. Ectasia was also seldom and identified in only 13% of thoracic arterial segments, of which the ascending aorta was preferentially involved. Despite a notable percentage of patients with periarterial thickening, increased vascular FDG uptake [Fig. 3A] was only demonstrated in 6/21 (29%) of patient's thoracic aortas and in 3/21 (14%) of patient's abdominal aortas.

Aortic arch branch vessel involvement was present in 26% of patients [Fig. 3B]. The left carotid was more commonly involved, followed by the innominate and left subclavian. Periarterial thickening was detected in 12% of left carotid, 11% subclavian and 10% innominate arteries. No occlusions were detected but stenosis was observed in 5% of left carotids, left subclavians, and innominates and 2% of right carotids and right subclavians, respectively.

Abnormalities in the visceral artery branches were present in 40% of patients, with the renal arteries most frequently involved (left 27%, right 18%) followed by the celiac artery (16%), superior mesenteric artery (SMA; 14%), and inferior mesenteric artery (IMA; 10%) [Fig. 3C]. In comparison to thoracic branch vessels, visceral arteries had a higher proportion of stenosis and occlusions. The left renal artery was stenotic in 10 (16%) patients and occluded in 2 (3%), whereas the right renal artery was stenotic in only 4 (6%) patients with no occlusions seen. Stenoses were also observed in the celiac artery (n = 4), SMA (n = 4) and IMA (n = 1) and occlusions detected in 1, 2, and 0 patients, respectively.

Iliofemoral arteries were affected in 19 (31%) patients and in keeping with other distributions had involvement with periarterial thickening as the most common finding [Fig. 3D]. Among patients with common iliac artery involvement, the majority had concomitant abnormalities in the infra-renal abdominal aorta. However, in 3 cases involvement of the common iliac arteries occurred in isolation of the abdominal aortic findings. Significantly fewer studies were available to evaluate the popliteal arteries. Only 1 patient had evidence of non-atherosclerotic occlusion of the right femoral and popliteal arteries in isolation of infra-renal aorta or iliac arterial pathologies. The remaining cases with popliteal findings had contiguous changes noted in the abdominal aorta that progressed distally. BRAF mutation was not associated with an increased likelihood of abnormalities in any particular arterial segment or group, including iliofemoral vessels.

Coronary artery and pulmonary artery findings were infrequently seen but limited due to lower number of dedicated coronary CTA, cardiac MRI, and conventional angiogram studies. Among available studies, periarterial thickening of the coronary arteries and pulmonary arteries were seen in 5% and 3% of cases, with stenoses (non-atherosclerotic) observed in 6 and 1 cases, respectively.

The presence of dense calcification denoting radiographic evidence of presumed atherosclerosis was seen in high frequency among multiple arterial segments at baseline (Table 2) with a severity and extent greater than anticipated for the patients’ age, lipid parameters and cardiovascular risk profile. Aortic segments (7%–46%) as well iliofemoral vessels (24%–45%) were noted to have the highest frequency of such findings [Fig. 3E].

## 7 Association between patient characteristics and arterial involvement at baseline

Patient characteristics, demographics, laboratory findings, presence/absence of cardiac involvement (excluding coronary atherosclerosis and coronary thickening) as well as perinephric involvement were assessed to determine their association with baseline arterial involvement. Increasing age was associated with a higher risk of thoracic aorta (odds ratio [OR], 95% confidence interval [CI]: 1.93 per 10 year increase [1.20, 3.10]; *P* = .007) and pelvic/lower extremity arterial involvement (OR [95% CI]: 1.73 [1.05, 2.85]; *P* = .032). The presence of diabetes (OR [95% CI]: 4.97 [1.19, 20.72]; *P* = .028) and hypertension (OR [95% CI]: 4.28 [1.33, 13.78]; *P* = .015) at baseline were associated with increased risk of visceral branch artery involvement.

Lower baseline hemoglobin was associated with a decreased risk of thoracic aorta (OR [95% CI]: 0.66 [0.47, 0.93]; *P* = .019) and abdominal aorta (OR [95% CI]: 0.66 [0.47, 0.92]; *P* = .015) abnormalities. Similarly, thoracic aorta (OR [95% CI]: 0.69 [0.49, 0.96]; *P* = .028) and abdominal aorta involvement (OR [95% CI]: 0.73 [0.55, 0.97]; *P* = .029) was less frequently detected among patients with elevated low-density lipoprotein (LDL) levels. The presence of fever at presentation was negatively associated with thoracic aorta (OR [95% CI]: 0.16 [0.03, 0.96]; *P* = .045) and pelvic/lower extremity (OR [95% CI]: 0.04 [0.00, 0.59]; *P* = .019) arterial abnormalities.

Perinephric fibrosis was strongly associated with the identification of abnormalities in the thoracic aorta (OR [95% CI]: 4.92 [1.54, 15.75]; *P* = .007), abdominal aorta (OR [95% CI]: 7.57 [2.28, 25.07]; *P* = .001) and visceral branch arteries (OR [95% CI]: 6.05 [1.52, 24.03]; *P* = .01) but not with the pelvic/lower extremity arteries. No significant associations among the variables investigated were observed to increase the likelihood of detecting aortic arch branch or coronary/pulmonary abnormalities at baseline.

## 8 Arterial interventions

A total of 8 patients underwent intervention with arterial endovascular stenting or bypass. One patient had a right coronary artery stent placed for angina before diagnosis and remained symptomatic with subsequent detection of ECD involving the coronary artery. Severe renovascular hypertension requiring stenting was present in 3 patients (1 bilateral, 2 unilateral). Lower extremity claudication symptoms resulted in endovascular stenting in 5 patients with the following distribution of stents: iliac (n = 2), femoral (n = 2), popliteal (n = 2), and tibioperoneal (n = 1) arteries. Only 1 patient underwent femoral-popliteal bypass.

## 9 Outcome

A total of 47 patients had follow-up imaging of arterial structures. The distributions of arterial findings at last follow-up are represented in Table 3. The majority of arterial segments affected at baseline did not have significant radiographic change during follow-up evaluation. Partial response was observed in 11% of patients with baseline abnormalities seen in the infra-renal abdominal aorta and 6% of patients with baseline aortic arch, descending thoracic aorta, and supra-renal abdominal aorta involvement, respectively. The remainder of arterial segments had partial response detected at a frequency of less than 5%.

**Table 3:**
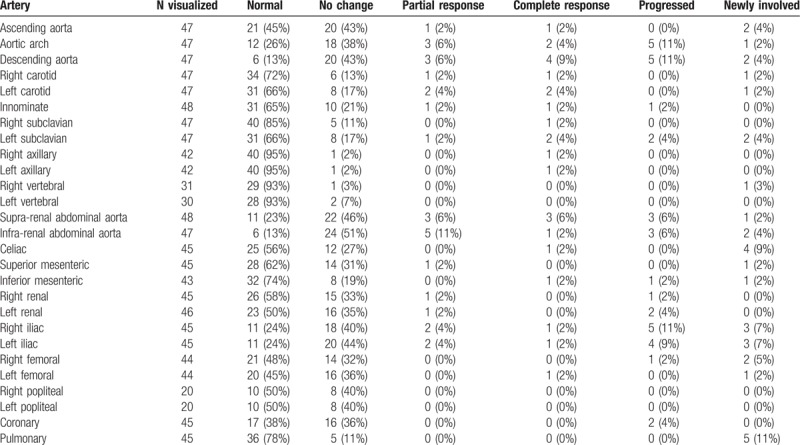
Outcome of arterial changes among patients with Erdheim–Chester disease with follow-up imaging.

Complete normalization of baseline arterial abnormalities was rare and observed only in 9% of patients with descending thoracic aorta and 6% supra-renal abdominal aorta involvement. The remainder of arterial segments had complete response in 4% or less of patients. Overall, progression of arterial abnormalities was uncommon but when present was most frequently detected among the aortic arch, descending thoracic aorta, supra and infra-abdominal aorta and bilateral iliac arteries. Development of arterial involvement after diagnosis in patients with normal baseline arteries was similarly infrequent but occurred most often in the pulmonary arteries (11%), celiac artery (9%), right iliac (7%), left iliac (7%), and right femoral (5%).

Treatment was not standardized and follow-up imaging studies were not systematically obtained before changing treatment regimens. Therefore, this study was not able to analyze the comparative effectiveness of specific medications and their impact on vascular outcomes. A description of the lines of treatment used in this cohort and the associated composite disease response is included in the supplementary materials (Table S1).

At last follow-up, 12 patients (19%) were deceased [Figure S1 and Table S2 in supplementary material]. Of the 9 patients for which cause of death was documented, only 3 patients died from cardiovascular causes, 2 of which were secondary to heart failure and 1 due to severe valvular heart disease.

## 10 Discussion

This report comprises the largest single-institution cohort of patients with ECD for which baseline and follow-up arterial studies were evaluated to determine the presence and evolution of aortic and branch vessel involvement.

In our series, aortic involvement occurred in 56% of patients evaluated with circumferential periarterial thickening the most common abnormality observed. Although case report reviews have described periaortic involvement as the presenting feature in as low as 1% of histologically proven cases with ECD,^[14]^ aortic involvement is often asymptomatic and therefore commonly under-reported.^[6]^ As such, unless thoracoabdominal imaging is performed, aortic and arterial findings are likely overlooked. Indeed the frequency of aortic infiltration in the current cohort is similar to comparable cohorts of similar size in which frequent or systematic imaging of the thorax was performed.^[5,6,8]^ However, despite the frequency of aortic infiltration, it does not appear that the structural integrity of the aortic wall is significantly impacted, as ectasia and aneurysm are uncommon in ECD at diagnosis or follow-up.

Although there appears to be a significant predilection for perivascular infiltration in the aortic wall, involvement of the aortic branches can also occur. A review of 66 case reports by Haroche and colleagues identified 13 cases (20%) which had main aortic branch vessel involvement documented.^[6]^ However, these authors suggested the true frequency of aortic branch vessel involvement is likely greater because all 6 of their additional cases had at least 1 main aortic branch involved by periarterial fibrosis, the most common locations being the SMA, left common carotid, and renal arteries.^[6]^ In a cohort of 40 ECD patients with thoracic CT scans, Brun and colleagues confirmed this suspicion, noting periarterial fibrotic changes could be seen in 73% of cases, with 60% of patients demonstrating findings in the supra-aortic trunk, 55% in the intercostal arteries, and 55% in coronary arteries.^[8]^ In our series, only 26% of patients had objective evidence of aortic arch branch involvement and 18% in coronary/pulmonary vessels. Whereas all patients in the Brun et al cohort had thoracic CT scans with intravenous contrast administered,^[8]^ this was not universal in our study and likely contributed to a lower detection rate in the arch branches and coronary arteries.

While abnormalities among the SMA, celiac, IMA, and renal arteries have been described,^[5,6,9]^ to date the frequency of visceral artery involvement in ECD has not been adequately characterized. In total, 40% of ECD patients in this study demonstrated abnormalities within visceral branch arteries. However, the majorities of these were asymptomatic and did not lead to mesenteric ischemia. Among patients with renal artery stenosis, severe renovascular hypertension was observed and when refractory to anti-hypertensives, briskly responded to endovascular stenting, as has been seen in other series.^[5,6]^

Limited information is currently present regarding iliofemoral and lower extremity artery involvement in ECD, in part because fewer dedicated scans are done to systematically evaluate these arterial segments. In addition, leg pain, if present, may be attributed to the highly frequent bone lesions observed in this condition and claudication may not be considered as an etiology. Furthermore, a review of symptoms and examination of lower extremity vessels appears to be lacking as the majority of patients in the present study did not have comprehensive detail regarding lower extremity vascular findings. Nevertheless, almost one-third of patients in our series had abnormalities in the iliofemoral and further distal arteries. Even though the majority of patients with iliofemoral findings had contiguous extension from the infra-renal abdominal aorta, involvement of the iliofemoral and popliteal vessels can occur in isolation of aortic findings. In contrast to the aortic arch and visceral branch vessels, surgical and endovascular interventions are more frequently required for claudication so a high-index of suspicion is necessary and a thorough lower extremity vascular examination is advocated in all patients at baseline and during follow-up. Investigation with non-invasive arterial studies utilizing duplex ultrasonography or CTA should be pursued if symptoms or examination abnormalities are present.

Due to the multisystem involvement, PET and PET-CT have been suggested as useful modalities for evaluating the distribution of lesions in ECD.^[15]^ In our series, baseline arterial hypermetabolism was seen in only 29% of patients with thoracic aortic involvement and 14% of patients with abdominal aortic involvement, demonstrating increased FDG-avidity on PET and/or PET-CT. Arnaud and colleagues noted similar discrepancies between PET and CT for detection of ECD involvement of large vessels at baseline with concordant findings in 15 of 24 patients, whereas CT detected large vessel abnormalities in 8 patients that did not have corresponding PET findings and only 1 patient had evidence of large vessel changes on PET that were not detected on CT.^[16]^ Therefore CT angiography may have additional utility in its ability to detect arterial involvement and should be included in the initial imaging evaluation when staging organ systems involved by ECD.

In addition to periarterial thickening, an interesting and under-reported finding observed in this cohort is the presence of calcified atheromatous disease. The frequency and severity of atherosclerotic changes in the abdominal and pelvic arteries appeared greater than anticipated based on the lipid profiles and cardiovascular risks. In particular, LDL levels were negatively associated with detection of ECD abnormalities in the thoracic and abdominal aorta. Although none of the vessels radiographically identified in our study were evaluated histologically to confirm the presence of atherosclerosis, severe atheromatous findings have been described in surgical and autopsy studies demonstrating dense yellow atherosclerotic plaque in the aorta and its branches of ECD patients.^[9,17–19]^ The mechanism of the accelerated atherosclerotic changes in ECD arteries is incompletely understood. However, it is known that lipid loading of macrophages through the enzyme acyl-coenzyme A:cholesterol-acyltransferase (ACAT) participates in the storage of lipids in the cytoplasm of arterial macrophages and their transition into arterial foam cells, which is integral in the development in atherosclerosis.^[20]^ In addition, cholesterol loading of macrophages can lead to foam cell secretion of pro-inflammatory cytokines interleukin (IL)-1, IL-6, tumor necrosis factor, and also chemokines CCL2 and CCL5.^[21]^ These similar cytokine and chemokines profiles have been noted to be upregulated in biopsies from lesioned tissue in ECD patients.^[22,23]^ Further research into the interplay between atherosclerosis and ECD is warranted.

In a large cohort of 165 ECD patients, Cohen-Aubart et al described a strongly positive correlation between the presence of BRAF mutation and detection of a right atrial pseudotumor (95% CI, 4.87–44.97, *P* < .0001).^[11]^ On the other hand, they did not observe BRAF mutation to correlate with pericardial involvement, coated aorta or other non-coronary vascular involvement. Our study similarly did not detect a significant association between BRAF mutation and aortic or vascular involvement. However, in contrast, we did not observe a correlation between BRAF mutation and right atrial pseudotumor. This lack of association is likely due to the limitation of a small proportion of patients in the present study tested for BRAF mutation in combination with the low frequency of detected right atrial abnormalities in our cohort.

Our study demonstrates that improvement of arterial abnormalities in patients with ECD is uncommon. Indeed, aside from limited improvement in 11% of infra-renal abdominal aortas, 6% or less of cases had partial improvement noted in the other areas of arterial abnormalities. Complete resolution of arterial findings is even more rarely observed. Although limited information is available in the literature regarding arterial outcomes, others have also noted that periaortic infiltration remains largely unchanged among smaller case series.^[6]^ FDG avidity may be more dynamic than arterial thickening but is still variable. Indeed while Arnaud and colleagues noted 33% of baseline FDG avid large vessel foci improved on repeat imaging with a variety of treatment modalities, 13% remained stable and 53% had an increased standardized uptake values (SUV) max at follow-up.^[16]^ Although the arterial distribution of progressive findings is not reported by Arnaud et al, the areas in our cohort that were most common to progress or become newly involved were the thoracic aorta, visceral and the iliac arteries. Although newly occurring lesions were uncommon, this highlights the need for comprehensive review of systems and examination in patients with ECD to elicit signs and symptoms of potential new arterial involvement.

This study must be interpreted in context of its limitations, including its retrospective design for which data obtained relies on documentation present in the medical record at the time of abstraction. In addition, while thoracoabdominal imaging was commonly obtained in the initial evaluation of patients with ECD, the timing and modality were not standardized. Furthermore, although contrast was commonly utilized, not all patients had arterial-phased contrast administration and thus subtle findings within aortic branch vessels may be under-reported. Because therapeutic regimens and timing of imaging were not standardized, we were not able to assess the impact of specific treatments on arterial outcomes. While 10 of the 12 patients with documented evidence of BRAF-V600E mutation received a targeted inhibitor (dabrafenib or vemurafenib), testing was only performed in one-third of patients in this cohort. Targeted BRAF inhibition has demonstrated effectiveness in ECD, particularly in central nervous system, ocular, kidney, bone, and skin infiltration,^[24]^ but detailed data showing evidence of arterial improvement is limited to a small open-label prospective study.^[25]^ Therefore, it is unknown if a higher proportion of patients in the current cohort would have demonstrated improvement in arterial outcomes if more frequent use of BRAF inhibitors was employed. Prospective studies with comprehensive arterial imaging are needed to determine the effect of targeted therapy in this disease.

In summary, aortic and aortic branch vessel abnormalities are frequently observed in patients with ECD and are often asymptomatic. Involvement of lower extremities may require vascular intervention and therefore focused examination should be incorporated into the baseline and follow-up evaluations. Improvement in periarterial thickening is uncommon during the course of disease. Progression or new involvement of the aorta and iliofemoral arteries can be observed; therefore ongoing evaluation of arterial structures in patients with ECD during follow-up should be incorporated into patient management strategies.

## Author contributions

**Conceptualization:** Michel Villatoro-Villar, Michael S. Bold, Kenneth J. Warrington, Cynthia S. Crowson, Gaurav Goyal, Mithun Shah, Ronald S. Go, Matthew J. Koster.

**Formal analysis:** Michel Villatoro-Villar, Michael S. Bold, Kenneth J. Warrington, Cynthia S. Crowson, Gaurav Goyal, Mithun Shah, Ronald S. Go, Matthew J. Koster.

**Investigation:** Michel Villatoro-Villar, Michael S. Bold, Kenneth J. Warrington, Cynthia S. Crowson, Gaurav Goyal, Mithun Shah, Ronald S. Go, Matthew J. Koster.

**Methodology:** Michel Villatoro-Villar, Michael S. Bold, Kenneth J. Warrington, Cynthia S. Crowson, Gaurav Goyal, Mithun Shah, Ronald S. Go, Matthew J. Koster.

**Validation:** Michel Villatoro-Villar, Michael S. Bold, Kenneth J. Warrington, Cynthia S. Crowson, Gaurav Goyal, Mithun Shah, Ronald S. Go, Matthew J. Koster.

**Writing – original draft:** Michel Villatoro-Villar, Michael S. Bold, Kenneth J. Warrington, Cynthia S. Crowson, Gaurav Goyal, Mithun Shah, Ronald S. Go, Matthew J. Koster.

**Writing – review & editing:** Michel Villatoro-Villar, Michael S. Bold, Kenneth J. Warrington, Cynthia S. Crowson, Gaurav Goyal, Mithun Shah, Ronald S. Go, Matthew J. Koster.

## Supplementary Material

Supplemental Digital Content
