# Supplementary material for: Arterial involvement in Erdheim–Chester disease: A retrospective cohort study
Source: Medicine (Baltimore). 2018 Dec 10;97(49):e13452. doi: 10.1097/MD.0000000000013452 (PMC6310516; doi:10.1097/MD.0000000000013452)
Supplement: Supplemental Digital Content [file medi-97-e13452-s001.docx]

**Supplementary Table 1: Individual treatments and response for cohort of patients with Erdheim-Chester disease**

| **Patient** | **Rx 1** | **Resp** | **Rx 2** | **Resp** | **Rx 3** | **Resp** | **Rx 4** | **Resp** | **Rx 5** | **Resp** | **Rx 6*** | **Resp** |
| --- | --- | --- | --- | --- | --- | --- | --- | --- | --- | --- | --- | --- |
|  |  |  |  |  |  |  |  |  |  |  |  |  |
| ECD-01 | GC | - | 2-CDA | - | - | - | - | - | - | - | - | - |
| ECD-02 | MTX | - | GC | - | ETN | NR | IFX | NR | ANA | - | 2-CDA | - |
| ECD-03 | RT | - | - | - | - | - | - | - | - | - | - | - |
| ECD-04 | IFX + MTX | - | Vem | - | - | - | - | - | - | - | - | - |
| ECD-05 | IFN-a | INT | 2-CDA | - | - | - | - | - | - | - | - | - |
| ECD-06 | - | - | - | - | - | - | - | - | - | - | - | - |
| ECD-07 | 2-CDA | - | - | - | - | - | - | - | - | - | - | - |
| ECD-08 | GC | - | CTX | - | - | - | - | - | - | - | - | - |
| ECD-09 | - | - | - | - | - | - | - | - | - | - | - | - |
| ECD-10 | Imatinib | PR | ANA | NR | Vem | - | - | - | - | - | - | - |
| ECD-11 | RT | NR | 2-CDA | NR | IFN-a | PR | - | - | - | - | - | - |
| ECD-12 | Renal Transplant | - | - | - | - | - | - | - | - | - | - | - |
| ECD-13 | 2-CDA | - | - | - | - | - | - | - | - | - | - | - |
| ECD-14 | GC + 2-CDA | - | - | - | - | - | - | - | - | - | - | - |
| ECD-15 | MTX + GC | NR | 2-CDA | NR | IFN-a | NR | - | - | - | - | - | - |
| ECD-16 | GC | NR | - | - | - | - | - | - | - | - | - | - |
| ECD-17 | IFN-a | - | - | - | - | - | - | - | - | - | - | - |
| ECD-18  ECD-19  ECD-20 | - | - | - | - | - | - | - | - | - | - | - | - |
|  | ANA | NR | IFN-a | INT | IFX + GC | INT | ANA + GC | - | - | - | - | - |
|  | GC | NR | MTX | NR | ANA | NR | Vem | PR | - | - | - | - |
| ECD-21 | MTX + GC | NR | IFN-a | - | - | - | - | - | - | - | - | - |
| ECD-22 | TAM | NR | - | - | - | - | - | - | - | - | - | - |
| ECD-23 | RT | NR | Vinb + GC | PR | RT | PR | - | - | - | - | - | - |
| ECD-24 | Vinb | NR | 2-CDA | NR | Samarium | PR | - | - | - | - | - | - |
| ECD-25 | GC | NR | CTX + GC | NR | GC | NR | MTX + GC | NR | IFN | NR | - | - |
| ECD-26 | PEG-IFN-a | PR | - | - | - | - | - | - | - | - | - | - |
| ECD-27 | PEG-IFN-a | PR | Vem | - | - | - | - | - | - | - | - | - |
| ECD-28 | 2-CDA | CR | GC | SD | - | - | - | - | - | - | - | - |
| ECD-29 | GC | PD | MTX + GC | PD | ANA | NR | IFN-a | INT | PEG-IFN-a | INT | Dabra+Tram | PR |
| ECD-30 | 2-CDA | SD | - | - | - | - | - | - | - | - | - | - |
| ECD-31 | - | - | - | - | - | - | - | - | - | - | - | - |
| ECD-32 | MTX | NR | IFX+ETN | NR | Vinb | NR | 2-CDA | SD | - | - | - | - |
| ECD-33 | 2-CDA | SD | - | - | - | - | - | - | - | - | - | - |
| ECD-34 | TAM+ GC | PR | CTX + GC | SD | - | - | - | - | - | - | - | - |
| ECD-35 | Vinb + GC | PR | 2-CDA | - | - | - | - | - | - | - | - | - |
| ECD-36 | GC | NR | AZA | NR | IFN-a | - | PEG-IFN-a | SD | - | - | - | - |
| ECD-37 | CTX | SD | - | - | - | - | - | - | - | - | - | - |
| ECD-38 | - | - | - | - | - | - | - | - | - | - | - | - |
| ECD-39 | GC | NR | MTX + IFX + GC | NR | Vem | SD | - | - | - | - | - | - |
| ECD-40 | Vem | CR | - | - | - | - | - | - | - | - | - | - |
| ECD-41 | 2-CDA | - | - | - | - | - | - | - | - | - | - | - |
| ECD-42 | GC | NR | MTX + GC | PR | - | - | - | - | - | - | - | - |
| ECD-43 | GC | NR | ANA | NR | Vem | INT | Dabra | PR | - | - | - | - |
| ECD-44 | MTX + IFX | SD | Vem | INT | IFX + MTX | SD | - | - | - | - | - | - |
| ECD-45 | DDAVP | - | GC | NR | RT | NR | GC | PR | 2-CDA | NR | - | - |
| ECD-46 | RT + GC | CR | - | - | - | - | - | - | - | - | - | - |
| ECD-47 | GC | PR | Ifos/Etop + GC | - | Cis | NR | - | - | - | - | - | - |
| ECD-48 | RT | CR | - | - | - | - | - | - | - | - | - | - |
| ECD-49 | - | - | - | - | - | - | - | - | - | - | - | - |
| ECD-50 | - | - | - | - | - | - | - | - | - | - | - | - |
| ECD-51 | GC | SD | MTX | NR | Dabra | - | ANA | - | Vem | - | - | - |
| ECD-52 | GC | NR | PEG-IFN-a | NR | 2-CDA | PR | - | - | - | - | - | - |
| ECD-53 | TAM | NR | TAM + GC | NR | AZA + GC + TAM | - | CTX + GC | PR | MTX | PR | - | - |
| ECD-54 | 2-CDA | SD | - | - | - | - | - | - | - | - | - | - |
| ECD-55 | ANA + MTX | PR | - | - | - | - | - | - | - | - | - | - |
| ECD-56 | CTX + GC | SD | - | - | - | - | - | - | - | - | - | - |
| ECD-57 | - | - | - | - | - | - | - | - | - | - | - | - |
| ECD-58 | GC | NR | - | - | - | - | - | - | - | - | - | - |
| ECD-59 | 2-CDA | NR | GC | NR | Ima | NR | - | - | - | - | - | - |
| ECD-60 | DDAVP | - | - | - | - | - | - | - | - | - | - | - |
| ECD-61 | GC | NR | AZA | SD | CTX | NR | GC | NR | - | - | - | - |
| ECD-62 | 2-CDA | CR | - | - | - | - | - | - | - | - | - | - |
| ECD-63 | Vem | PR | 2-CDA | PR | - | - | - | - | - | - | - | - |
| ECD-64 | PEG-IFN-a | - | Vem | - | - | - | - | - | - | - | - | - |
| 2-CDA, cladribine; ANA, anakinra; AZA, azathioprine; Cis, cisplatin; CR, complete response; CTX, cyclophosphamide; Dabra, dabrafenib; DDAVP, desmopressin; ECD: Erdheim-Chester disease; ETN, etanercept; Etop, etoposide; GC, glucocorticoids; Ifos, ifosfamide; IFN-a, interferon-alpha; IFX, infliximab; Ima, Imatinib; INT, intolerance; MTX, methotrexate; NR, non-response, PEG-IFN-a, pegylated interferon-alpha; Rx: treatment; Resp: response; GC: glucocorticoids; MTX: methotrexate; RT: radiotherapy; IFX: infliximab; IFN-a: interferon-alpha; 2-CDA: cladribine; Ima: imatinib; Vinb: vinblastin; PD, progression of disease; PEG-IFN-a: pegylated interferon-alpha; PR, partial response; Resp, response; RT, radiotherapy; Rx, treatment; SD, stable disease; Tram, trametinib; Vem, vemurafenib; Vinb, vinblastine.  *After the 6^th^ line of treatment Patient ECD-02 underwent surgical excision followed by successive rounds of canakinumab and tocilizumab | | | | | | | | | | | | |

**Supplementary Figure 1: Overall survival**

Legend

Overall survival related to Erdheim-Chester disease compared to expected rates from US white lifetables; observed: solid line; expected dashed line.


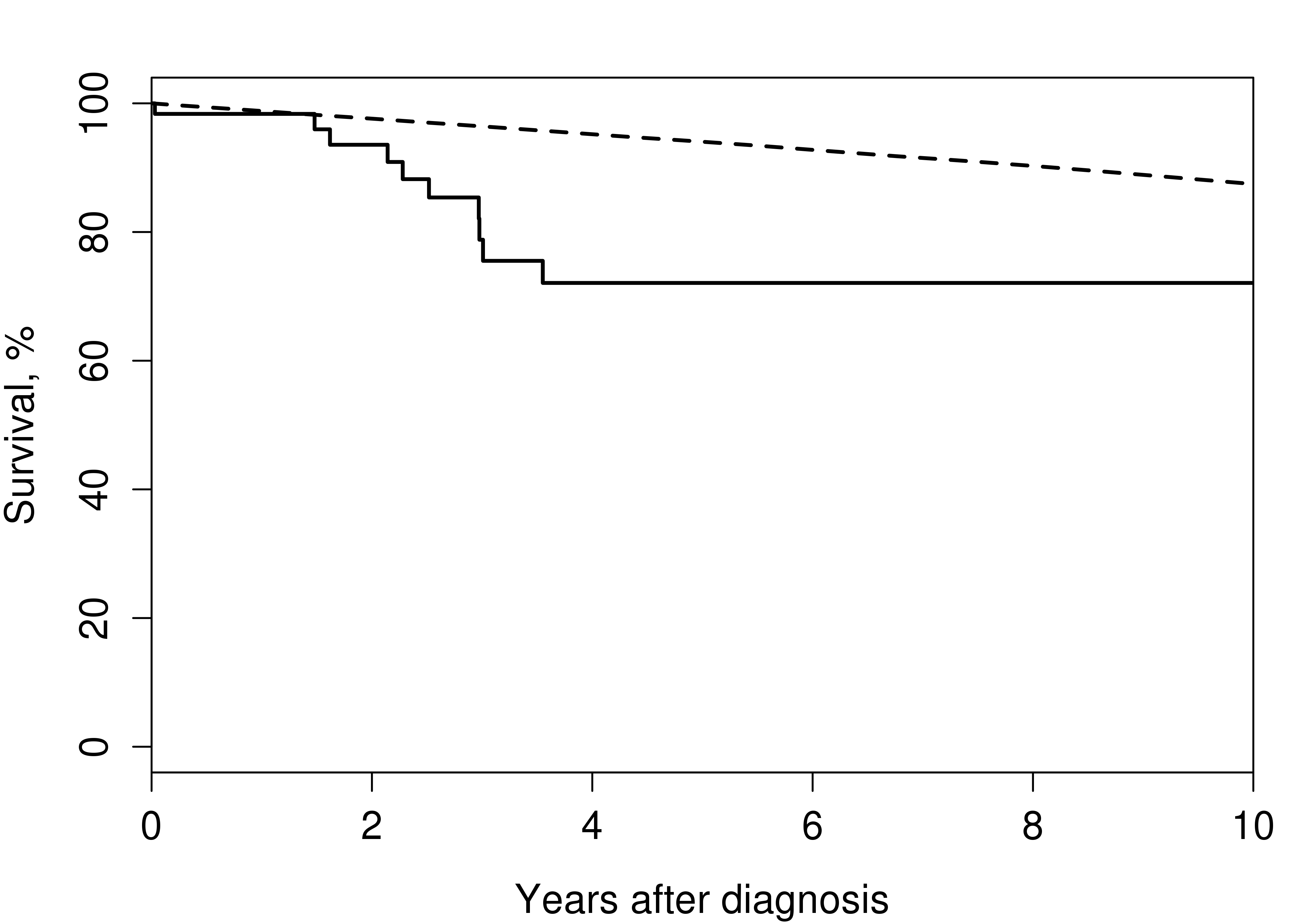


**Supplementary Table 2: Survival rates**

| **Measure** | **Overall** |
| --- | --- |
| Number of patients | 64 |
| Number of deaths | 12 |
| Expected number of deaths | 3.1 |
| Standardized mortality ratio (95% CI) | 3.91 (2.02, 6.84) |
| 1-sample log rank test p-value | <0.001 |
| 1 year survival rate (95% CI) | 98 (95, 100) |
| 2 year survival rate (95% CI) | 94 (87, 100) |
| 5 year survival rate (95% CI) | 72 (58, 89) |
| 10 year survival rate (95% CI) | 72 (58, 89) |

CI=confidence interval.
